# Risk of Death and Cardiovascular Outcomes with Thiazolidinediones: A Study with the General Practice Research Database and Secondary Care Data

**DOI:** 10.1371/journal.pone.0028157

**Published:** 2011-12-02

**Authors:** Arlene M. Gallagher, Liam Smeeth, Suzie Seabroke, Hubert G. M. Leufkens, Tjeerd P. van Staa

**Affiliations:** 1 General Practice Research Database, Medicines and Healthcare products Regulatory Agency, London, United Kingdom; 2 Utrecht Institute for Pharmaceutical Sciences, Utrecht University, Utrecht, The Netherlands; 3 London School of Hygiene & Tropical Medicine, London, United Kingdom; 4 Division of Vigilance and Risk Management of Medicines, Medicines and Healthcare products Regulatory Agency, London, United Kingdom; Universidad Peruana Cayetano Heredia, Peru

## Abstract

**Objective:**

To describe the likely extent of confounding in evaluating the risks of cardiovascular (CV) events and mortality in patients using diabetes medication.

**Methods:**

The General Practice Research Database was used to identify inception cohorts of insulin and different oral antidiabetics. An analysis of bias and incidence of mortality, acute coronary syndrome, stroke and heart failure were analysed in GPRD, Hospital Episode Statistics and death certificates.

**Results:**

206,940 patients were identified. The bias analysis showed that past thiazolidinedione users had a lower mortality risk compared to past metformin users. There were no differences between past users of rosiglitazone and pioglitazone (adjusted RR of 1.04; 95% CI 0.93–1.18). Current rosiglitazone users had an increased risk of death (adjusted RR 1.20; 95% CI 1.08–1.34) and of hospitalisation for heart failure (adjusted RR of 1.73; 95% CI 1.19–2.51) compared to current pioglitazone users. Risk of mortality was increased two-fold shortly after starting rosiglitazone. Excess risk of death over 3 years with rosiglitazone was 0.3 per 100 in those aged 50–64 years, 2.0 aged 65–74, 3.0 aged 75–84, and 7.0 aged 85+. The cause of death with rosiglitazone was more likely to be due to a disease of the circulatory system.

**Conclusions:**

Higher risks for death (overall and due to cardiovascular disease) and heart failure were found for rosiglitazone compared to pioglitazone. These excess risks were largest in patients aged 65 years or older. The European regulatory decision to suspend rosiglitazone is supported by this study.

## Introduction

The thiazolidinediones - rosiglitazone and pioglitazone - have been widely used for the treatment of type 2 diabetes mellitus. However, signals concerning the cardiovascular safety have been emerging over the last few years. An increased risk of myocardial infarction (MI), with no increases in mortality was found for rosiglitazone in a meta-analysis of randomised controlled trials (RCTs) [1], [2]. Pioglitazone was reported to have a statistically significant lower risk in a composite endpoint of death, MI and stroke from findings of another meta-analysis [3]. These reports were supported by a FDA meta-analysis of RCTs, which found that the risk of all-cause and cardiovascular mortality and MI tended to be lower with pioglitazone and higher with rosiglitazone (although most results did not reach statistical significance) [4]. This analysis also highlighted that the risk of congestive heart failure was increased with both drugs [4]. Another study also noted the increase in numbers of congestive heart failure with both thiazolidinediones [5]. Limitations of these meta-analyses are the low number of events and the pooling of studies with varying designs and study populations. Furthermore, the populations using the drugs and exposure characteristics in actual clinical practice may be different from those enrolled in RCTs [6].

A large number of observational studies have also evaluated the cardiovascular safety of rosiglitazone or pioglitazone but the quality of these studies varied considerably [7]–[32]. An unpublished assessment by the UK medicines and medical devices regulatory authority of 24 observational studies noted substantial limitations in most studies, including limited statistical power, short durations of follow-up or study populations with lack of long-term continuity of data collection. Also, observational studies relied on the power of statistical adjustment to deal with confounding. Confounding (which is a challenge in any observational study) may be even more challenging in studies of diabetes, since drug exposure is defined by diabetes severity, making it very difficult to separate the effect of disease severity from treatment.

There has been an ongoing European regulatory review of the cardiovascular safety of thiazolidinediones and a study was commissioned by the UK Medicines and Healthcare products Regulatory Agency (MHRA) (this was done prior to the subsequent suspension of rosiglitazone in Europe). The first objective of the present study was to describe the likely extent of confounding in evaluating the risks of cardiovascular events and mortality in patients using diabetes medication. Where confounding effects could be adequately controlled, the second objective was to compare the risks of cardiovascular events and mortality between different types of diabetes medication, including rosiglitazone and pioglitazone.

## Methods

### Data source

This study used data from the General Practice Research Database (GPRD) in the United Kingdom. GPRD comprises the computerised medical records maintained by general practitioners (GPs). GPs play a key role in the UK health care system, as they are responsible for primary health care and specialist referrals. Patients are affiliated with a practice, which centralises the medical information from the GPs, specialist referrals and hospitalisations. The data recorded in the GPRD since 1987 include demographic information, prescription details, clinical events, preventive care provided, specialist referrals, hospital admissions and their major outcomes [33]. A recent review of all validation studies found that medical data in GPRD were generally of high quality [34]. Patients in about 40% of GPRD have now been linked individually and anonymously to the national registry of hospital admission (Hospital Episode Statistics [HES]) and to the death certificates (as collected by the Office of National Statistics). For each hospitalised patient, the hospital charts are reviewed, dates of admission and discharge and main diagnoses are extracted, coded by coding staff and collated nationally into HES. The death certificates list the date and causes of death. HES data were available from April 1997 and death certificates from January 2001 for about 40% of GPRD practices. The data from HES and GPRD were recorded and collected independently from each other.

### Study population

The exposed study cohort consisted of adults aged 40 years and older with a prescription for insulin or oral antidiabetic drugs (OAD) at least one year after start of data collection. Patients with a record of type I diabetes were excluded. The index date was the first prescription for insulin or OAD one year after start of GPRD data collection (the prescribing prior to the index date was not considered in the creation of this prevalent user cohort). The period of follow-up was from the index date up to the date of censoring (i.e., transfer out of the practice, last collection from the practice, or death). Each exposed patient was matched by age (within 5 years), sex and practice to one control patient, with the index date of the control being the same as that of the exposed patient. Within this overall exposed cohort, we identified inception cohorts for each class of diabetes medication (a patient was included in an inception cohort if they received first-ever prescription for a class of diabetes medication at least one year after start of GPRD data collection). The medications of interest in this study were thiazolidinediones, insulins, metformin and sulphonylureas. Furthermore, we also created two inception cohorts separately for pioglitazone and rosiglitazone. Patients prescribed multi-constituent preparations were included in multiple classes of diabetes medication.

In order to prevent immortal time bias and incorrect exclusion of patients based on events that occur after the index date, patients could belong to multiple inception cohorts. In comparisons between different diabetes medications, patients were censored at the start of treatment with the medication of the reference group. The exposure to each diabetes medication was classified in a time-dependent manner, dividing the period of follow-up into periods of current, recent and past exposure. The period of current exposure was defined as the period from the date of a prescription up to 3 month after the date of the prescription. Recent use was the period of time from 3 to 12 months after the most recent prescription and past use was the time from 12 months after. Patients could move between exposure categories over time.

### Outcomes of interest

The following incident outcomes were measured: death due to any cause (as recorded in GPRD or on death certificates), cause of death (death certificates), acute coronary syndrome (ACS) (recorded in GPRD or HES), stroke (GPRD or HES) and heart failure (GPRD or HES). Analyses requiring the HES or ONS data were restricted to patients from practices participating in the linkage and to those with data during the HES/ONS data collection period. Given the different coding dictionaries used by the various datasets and different methods for data collection, outcomes from each source were analysed separately.

### Statistical analyses

Four sets of analyses were conducted. The first set of analyses evaluated the possible extent of bias in the comparisons between different diabetes medications and whether statistical adjustment with risk factors would sufficiently address any confounding. In this analysis, the incidence of various outcomes was compared between past exposure for each of the diabetes medications and matched control patients. Poisson regression was used to estimate relative rates (RRs). These models also included age, sex, calendar year, small-area socioeconomic status (for linked practices), smoking status, use of alcohol, body mass index, medical history ever before of coronary heart disease, coronary revascularisation, hyperlipidaemia, hypertension, peripheral vascular disease, renal impairment and stable angina and prescribing in the 6 months before of angiotensin II receptor blockers, antiplatelets, beta blockers, calcium channel blockers, diuretics, nitrates, NSAIDs or aspirin and statins. In addition, the models included use of current use of the various classes of diabetes medication. Missing values for alcohol use, smoking status and body mass index were included as separate categories in the regression analyses. This bias analysis explored whether statistical adjustment substantially reduced the point estimates of RRs towards one. If the adjusted RRs would remain elevated, this could either indicate residual confounding (i.e., effects of the underlying disease) or persistent adverse effects after treatment discontinuation.

The second set of analyses concerned a comparison of the rates of outcomes during current use of different diabetes medications. These analyses were restricted to the types of diabetes medications that did not have major differences in risk during past use (as observed in the previous set of analyses). These analyses were stratified by age, co-prescribing of insulin and calendar time (before and after 2007; in October 2007 additional warnings for cardiovascular disease with rosiglitazone were communicated by the UK regulatory authority.

The third set of analyses described the pattern of risks over duration of treatment. The follow-up period of current exposure was divided into 100 periods and the absolute risk was estimated within each small period. These estimates were then smoothed using the method proposed by Ramlau-Hansen [35]. This analysis of hazard rates displays visually the observed (crude) risks over duration of current exposure to a diabetes medication.

The last set of analyses estimated the cumulative incidence over time with current use of various medications for diabetes. Kaplan-Meier life-tables were estimated. These life-tables describe the absolute incidence over time, accounting for loss to follow-up while not adjusting for any risk factors.

## Results

### Demographics

The overall study population included 206,940 patients prescribed insulin or OAD and the same number of controls without diabetes. Table 1 shows the baseline characteristics of the inception cohorts of metformin, sulphonylureas, rosiglitazone, pioglitazone and insulins. As expected, there were differences in risk factors between metformin, sulphonylureas and insulin (metformin was more often the first diabetes treatment while insulin users had more frequent history of use of other diabetes treatments). There were no major differences at baseline for most characteristics between rosiglitazone and pioglitazone, except for prescribing over calendar time and higher prior use of statins among pioglitazone users (this was found to be related to secular changes in statin prescribing). The number of patients starting rosiglitazone dropped substantially after 2007 (2006, N = 4583; 2007, N = 2612; 2008, N = 474; 2009, N = 278). The use of pioglitazone changed differently (2006, N = 1543; 2007, N = 2815; 2008, N = 4660; 2009, N = 3177).

**Table 1:** Baseline characteristics at inception date of metformin, sulphonylureas, rosiglitazone, pioglitazone and insulin.

Characteristic	Metformin N = 121,637 N (%)	Sulphonylureas N = 76,863 N (%)	Rosiglitazone N = 22,636 N (%)	Pioglitazone N = 18,953 N (%)	Insulin N = 26,458 N (%)
Mean Age, years (sd)	64 (12)	66 (12)	63 (11)	64 (11)	66 (11)
Median Age, years (IQR)	64 (55–73)	66 (57–74)	63 (55–72)	64 (56–72)	66 (57–74)
Sex: Female	53,649 (44.1%)	33,982 (44.2%)	9,939 (43.9%)	7,939 (41.9%)	11,678 (44.1%)
Male	67,988 (55.9%)	42,881 (55.8%)	12,697 (56.1%)	11,014 (58.1%)	14,780 (55.9%)
Mean Follow-up, years (sd)	4 (3)	5 (4)	5 (2)	3 (2)	4 (3)
Mean BMI (sd)	31 (6)	30 (6)	31 (6)	32 (6)	30 (6)
Mean HbA1c,% (sd)	8.8 (1.8)	9.0 (1.8)	8.9 (1.5)	8.7 (1.5)	9.8 (1.8)
Smoking Status Non Smoker	48,487 (39.9%)	30,748 (40.0%)	8,736 (38.6%)	6,799 (35.9%)	9,941 (37.6%)
Ex Smoker	46,146 (37.9%)	26,809 (34.9%)	9,556 (42.2%)	8,989 (47.4%)	10,421 (39.4%)
Smoker	22,330 (18.4%)	13,760 (17.9%)	3,950 (17.5%)	3,031 (16.0%)	4,909 (18.6%)
Unknown	4,674 (3.8%)	5,546 (7.2%)	394 (1.7%)	134 (0.7%)	1,187 (4.5%)
Hospitalisation in the year before	11,391 (22.6%)	8,658 (27.4%)	1,979 (20.5%)	1,747 (22.0%)	5,112 (43.8%)
Number of diabetes medication classes ever before: 0	89,922 (73.9%)	36,260 (47.2%)	645 (2.8%)	389 (2.1%)	2,917 (11.0%)
1	29,067 (23.9%)	34,393 (44.7%)	8,628 (38.1%)	4,595 (24.2%)	3,081 (11.6%)
2	2,371 (1.9%)	5,703 (7.4%)	11,302 (49.9%)	8,747 (46.2%)	11,024 (41.7%)
3	248 (0.2%)	459 (0.6%)	1,838 (8.1%)	4,357 (23.0%)	7,683 (29.0%)
4	29 (0.0%)	45 (0.1%)	207 (0.9%)	746 (3.9%)	1,563 (5.9%)
5+	0 (0.0%)	3 (0.0%)	16 (0.1%)	119 (0.6%)	190 (0.7%)
History of ACS	13,132 (10.8%)	9,017 (11.7%)	2,378 (10.5%)	1,989 (10.5%)	5,056 (19.1%)
Stroke	8,031 (6.6%)	5,944 (7.7%)	1,349 (6.0%)	1,172 (6.2%)	2,534 (9.6%)
Heart failure	5,294 (4.4%)	5,268 (6.9%)	831 (3.7%)	550 (2.9%)	2,765 (10.5%)
Stable angina	15,900 (13.1%)	11,009 (14.3%)	3,081 (13.6%)	2,511 (13.2%)	4,903 (18.5%)
Hyperlipidaemia	11,488 (9.4%)	6,685 (8.7%)	2,791 (12.3%)	2,572 (13.6%)	2,925 (11.1%)
Hypertension	84,968 (69.9%)	53,462 (69.6%)	17,618 (77.8%)	15,424 (81.4%)	20,470 (77.4%)
Recent prescribing of Nitrates	11,664 (9.6%)	8,540 (11.1%)	2,158 (9.5%)	1,653 (8.7%)	4,308 (16.3%)
Beta blockers	29,206 (24.0%)	17,938 (23.3%)	5,636 (24.9%)	4,623 (24.4%)	7,065 (26.7%)
Calcium channel blockers	29,382 (24.2%)	18,532 (24.1%)	6,183 (27.3%)	5,681 (30.0%)	7,656 (28.9%)
Diuretics	41,234 (33.9%)	27,929 (36.3%)	7,651 (33.8%)	6,645 (35.1%)	10,917 (41.3%)
Antiplatelets	43,468 (35.7%)	27,971 (36.4%)	10,441 (46.1%)	10,049 (53.0%)	12,669 (47.9%)
ACE inhibitors/angiotensin II receptor blockers	51,946 (42.7%)	31,870 (41.5%)	13,132 (58.0%)	12,371 (65.3%)	14,595 (55.2%)
Statins or fibrates	61,239 (50.3%)	35,295 (45.9%)	15,271 (67.5%)	14,934 (78.8%)	15,502 (58.6%)
NSAIDs	54,420 (44.7%)	34,424 (44.8%)	12,111 (53.5%)	11,071 (58.4%)	14,010 (53.0%)

### Bias analyses

Table 2 shows the results of the bias analyses. The statistical comparison showed major differences, as expected, in the risks of cardiovascular outcomes and death between the overall exposed cohort and control cohort. Statistical adjustment did reduce the RRs for the cardiovascular outcomes, although statistically significant differences remained. For mortality, statistical adjustment increased the RRs with higher risks in the exposed cohort (indicating that diabetes treatments are less likely to be given to patients at imminent risk of death). In the analysis of past exposure, it was found that patients who had discontinued insulin had a higher risk of death compared to past users of metformin. Past thiazolidinediones users had a lower risk of death. The RRs were comparable in past rosiglitazone users compared to past pioglitazone users (adjusted RRs of 1.04 [95% 0.93–1.18], 1.11 [95% CI 0.86–1.43], 0.76 [95% CI 0.56–1.03] and 0.95 [95% CI 0.74–1.23 for, respectively, death, ACS, stroke and heart failure in GPRD). These results suggest that comparisons of different classes of diabetes medications are likely to be prone to substantial confounding, while the within class comparison of rosiglitazone versus pioglitazone is less prone to selection bias and confounding.

**Table 2:** Rates of outcomes (in GPRD) during past exposure compared to matched control cohort or to past metformin exposure.

		Comparison with control cohort without diabetes		Comparison with past exposure of metformin	
Outcome	Drug Class	Age, sex, calendar year adjusted RR (95%CI)	Fully adjusted RR (95%CI)	Age, sex, calendar year adjusted RR (95%CI)	Fully adjusted RR (95%CI)
Death	Insulin	2.40 (2.20–2.61)	2.84 (2.60–3.11)	1.38 (1.26–1.50)	1.24 (1.12–1.37)
	Sulphonylureas	1.77 (1.72–1.82)	2.18 (2.11–2.26)	0.96 (0.89–1.03)	0.97 (0.90–1.04)
	Thiazolidinediones	1.77 (1.66–1.88)	3.04 (2.77–3.33)	0.81 (0.76–0.85)	0.84 (0.79–0.89)
	Metformin	2.01 (1.96–2.07)	2.23 (2.15–2.31)	Reference	Reference
	Controls	Reference	Reference	N/A	N/A
ACS	Insulin	2.00 (1.54–2.61)	1.68 (1.27–2.21)	1.00 (0.76–1.32)	1.04 (0.79–1.37)
	Sulphonylureas	2.38 (2.23–2.54)	1.63 (1.51–1.77)	1.23 (1.03–1.48)	1.29 (1.08–1.55)
	Thiazolidinediones	2.47 (2.17–2.81)	1.61 (1.31–1.97)	1.03 (0.91–.118)	1.05 (0.92–1.20)
	Metformin	2.48 (2.32–2.67)	1.55 (1.42–1.68)	Reference	Reference
	Controls	Reference	Reference	N/A	N/A
Stroke	Insulin	1.91 (1.45–2.52)	1.91 (1.43–2.56)	0.99 (0.75–1.32)	0.95 (0.71–1.26)
	Sulphonylureas	1.82 (1.68–1.97)	1.60 (1.45–1.76)	1.01 (0.82–1.25)	1.04 (0.85–1.29)
	Thiazolidinediones	1.90 (1.61–2.25)	1.89 (1.46–2.45)	0.91 (0.77–1.07)	0.92 (0.79–1.09)
	Metformin	2.05 (1.89–2.22)	1.72 (1.56–1.89)	Reference	Reference
	Controls	Reference	Reference	N/A	N/A
Congestive Heart Failure	Insulin	2.23 (1.74–2.87)	1.62 (1.25–2.10)	0.94 (0.73–1.22)	0.94 (0.73–1.22)
	Sulphonylureas	2.45 (2.30–2.62)	1.42 (1.31–1.54)	1.20 (0.99–1.44)	1.34 (1.12–1.62)
	Thiazolidinediones	3.23 (2.80–3.72)	1.75 (1.41–2.17)	1.12 (0.98–1.28)	1.13 (0.99–1.29)
	Metformin	2.97 (2.77–3.19)	1.50 (1.38–1.63)	Reference	Reference
	Controls	Reference	Reference	N/A	N/A

### Comparison of rates of outcomes with current diabetic medications

As shown in Table 3, current rosiglitazone users had an increased risk of death compared to current pioglitazone users (adjusted RR 1.20 [95% CI 1.08–1.34]). The rates of ACS and stroke were comparable between rosiglitazone and pioglitazone. Hospital admission for congestive heart failure was increased with rosiglitazone (adjusted RR of 1.73 [95% CI 1.19–2.51]) while heart failure diagnosed or treated by a GP was statistically comparable between the two types of thiazolidinediones (adjusted RR of 1.14 [95% CI 0.97–1.34]). Table 4 shows the analyses stratified by age, co-prescribing of insulin and calendar time. The RRs comparing current rosiglitazone to pioglitazone users did not vary substantially.

**Table 3:** Rates of outcomes during current exposure of pioglitazone and rosiglitazone.

Outcome	Class	Number of cases	Incidence rate&	Age, sex, calendar year adjusted RR(95% CI)	Fully adjusted RR(95%CI)
Death (GPRD)	Pioglitazone	487	1.7	Reference	Reference
	Rosiglitazone	1274	2.0	1.15 (1.04–1.28)	1.20 (1.08–1.34)
Death (ONS)	Pioglitazone	145	1.6	Reference	Reference
	Rosiglitazone	469	1.9	1.09 (0.90–1.32)	1.07 (0.89–1.29)
ACS (GPRD)	Pioglitazone	219	0.9	Reference	Reference
	Rosiglitazone	510	0.9	0.99 (0.84–1.16)	1.03 (0.87–1.21)
ACS (HES)	Pioglitazone	67	0.8	Reference	Reference
	Rosiglitazone	203	0.9	1.04 (0.79–1.37)	1.02 (0.77–1.35)
Stroke (GPRD)	Pioglitazone	108	0.4	Reference	Reference
	Rosiglitazone	254	0.4	1.00 (0.80–1.26)	1.05 (0.83–1.32)
Stroke (HES)	Pioglitazone	31	0.4	Reference	Reference
	Rosiglitazone	105	0.4	1.16 (0.77–1.74)	1.12 (0.75–1.68)
Congestive Heart Failure (GPRD)	Pioglitazone	208	0.7	Reference	Reference
	Rosiglitazone	534	0.9	1.08 (0.92–1.27)	1.14 (0.97–1.34)
Congestive Heart Failure (HES)	Pioglitazone	34	0.4	Reference	Reference
	Rosiglitazone	172	0.7	1.73 (1.19–2.50)	1.73 (1.19–2.51)

& Number of cases per 100 person years.

**Table 4:** Rates of outcomes (in GPRD) during current exposure of rosiglitazone compared to pioglitazone stratified by age, co-prescribing of insulin and calendar time.

Outcome	Stratification	Age, sex, calendar year adjusted RR (95%CI)	Fully adjusted RR (95%CI)
Death	Age <65	1.08 (0.82–1.41)	1.14 (0.87–1.49)
	≥65	1.17 (1.04–1.31)	1.22 (1.09–1.37)
ACS	Age <65	0.79 (0.61–1.03)	0.82 (0.64–1.07)
	≥65	1.12 (0.92–1.38)	1.17 (0.95–1.43)
Stroke	Age <65	0.84 (0.52–1.36)	0.90 (0.56–1.46)
	≥65	1.05 (0.81–1.36)	1.09 (0.84–1.41)
Congestive Heart Failure	Age <65	0.90 (0.61–1.34)	0.95 (0.64–1.42)
	≥65	1.12 (0.94–1.34)	1.19 (0.99–1.42)
Death	Co-prescribing insulin: no	1.16 (1.05–1.30)	1.22 (1.09–1.35)
	yes	1.12 (0.63–1.97)	1.20 (0.68–2.13)
ACS	Co-prescribing insulin: no	0.98 (0.83–1.15)	1.00 (0.85–1.18)
	yes	1.70 (0.71–4.08)	1.83 (0.76–4.42)
Stroke	Co-prescribing insulin: no	0.98 (0.78–1.23)	1.02 (0.81–1.29)
	yes	2.54 (0.49–13.16)	2.66 (0.51–13.84)
Congestive Heart Failure	Co-prescribing insulin: no	1.11 (0.94–1.31)	1.16 (0.98–1.37)
	yes	0.73 (0.31–1.75)	0.80 (0.33–1.91)
Death	<2007 (calendar year)	1.15 (0.98–1.35)	1.20 (1.03–1.41)
	≥2007	1.13 (0.98–1.30)	1.19 (1.03–1.37)
ACS	<2007	1.04 (0.83–1.30)	1.06 (0.84–1.33)
	≥2007	0.90 (0.71–1.13)	0.94 (0.75–1.19)
Stroke	<2007	1.33 (0.94–1.87)	1.40 (0.99–1.98)
	≥2007	0.74 (0.54–1.02)	0.76 (0.55–1.06)
Congestive Heart Failure	<2007	1.04 (0.83–1.29)	1.10 (0.88–1.37)
	≥2007	1.13 (0.89–1.43)	1.20 (0.94–1.53)

Table 5 shows the mortality rates and primary cause of death in current rosiglitazone and pioglitazone users. Rosiglitazone users were more likely to die due to a disease of the circulatory system compared to pioglitazone users (although these findings did not reach statistical significance).

**Table 5:** Mortality rates and primary cause of death in rosiglitazone and pioglitazone current users.

	Rosiglitazone		Pioglitazone		
Primary cause of death (ICD 10 codes)	Number of cases	Incidence rate&	Number of cases	Incidence rate&	Age, sex, calendar year adjusted RR (95% CI)
All cause	469	1.9	145	1.63	1.09 (0.90–1.32)
A00–B99: Certain infectious and parasitic diseases	7	0.03	7	0.08	0.30 (0.10–0.87)
C00–D89: Neoplasms/Diseases of the blood and blood forming organs	106	0.43	38	0.43	0.95 (0.65–1.38)
E00–E90: Endocrine, nutritional and metabolic disorders	25	0.1	14	0.16	0.56 (0.29–1.09)
F00–F99: Mental and behavioural disorders	3	0.01	1	0.01	1.30 (0.13–12.77)
G00–G99: Diseases of the nervous system	1	0	3	0.03	0.11 (0.01–1.05)
I00–I99: Diseases of the circulatory system	223	0.9	56	0.63	1.34 (1.00–1.80)
I20–I25: Ischemic heart disease	176	0.71	47	0.53	1.26 (0.91–1.75)
I50: Heart failure	88	0.36	21	0.24	1.36 (0.84–2.19)
J00–J99: Diseases of the respiratory system	55	0.22	10	0.11	1.91 (0.97–3.77)
K00–K93: Diseases of the digestive system	25	0.1	11	0.12	0.78 (0.38–1.59)
L00–L99: Diseases of the skin and subcutaneous tissue	4	0.02	0	0	
M00–M99: Diseases of the musculoskeletal system and connective tissue	1	0	1	0.01	0.30 (0.02–5.00)
N00–N99: Diseases of the genitourinary system	8	0.03	1	0.01	2.43 (0.30–19.57)
V01–Y98: External causes of morbidity and mortality	9	0.04	3	0.03	1.10 (0.29–4.12 )

& Number of cases per 100 person years.

### Patterns of Risks

**Figure 1:**
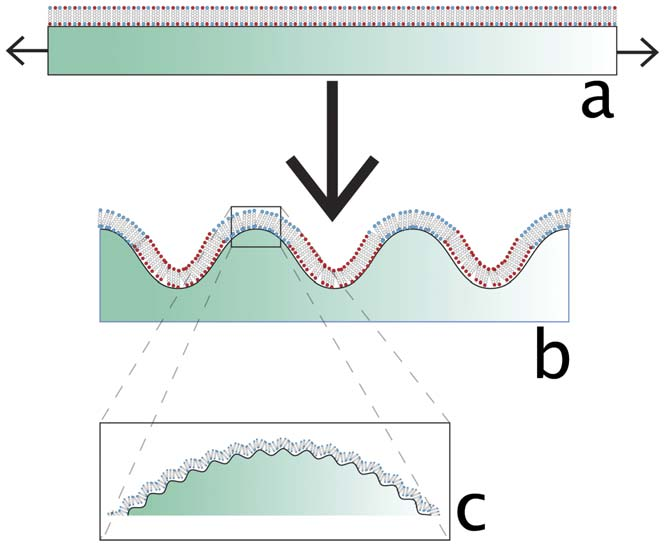
Smoothed crude RR of death over duration of treatment (years) in rosiglitazone users compared to pioglitazone users. RR: relative rate.

Figure 1 shows the smoothed RRs over duration of treatment in rosiglitazone users compared to pioglitazone users. It was found that the risk of mortality was increased about two-fold shortly following the start of rosiglitazone treatment (compared to pioglitazone) and then remained elevated (although at a much lower level) with longer treatment duration.

### Cumulative incidence over time

An additional seven patients (per 100 patients over 3 years) died during rosiglitazone treatment compared to pioglitazone in those aged 85 years or older (Table 6). The excess risks were progressively smaller in younger patients (there was an excess risk of death of 0.3 per 100 in those aged 65 years or younger). Appendix S1 lists the cumulative incidence of ACS, stroke and heart failure.

**Table 6:** Cumulative incidence (%) of outcomes (in GPRD) over one and three years in current rosiglitazone and pioglitazone users.

			Year 1			Year 3		
Outcome	Strata		Rosiglitazone	Pioglitazone	Excess risk	Rosiglitazone	Pioglitazone	Excess risk
Death	All		1.85 (1.65–2.06)	1.48 (1.25–1.76)	0.36	5.80 (5.37–6.27)	4.46 (3.89–5.11)	1.35
	Age	40–49	0.21 (0.08–0.57)	0.12 (0.02–0.85)	0.09	0.82 (0.44–1.50)	0.56 (0.21–1.50)	0.25
		50–64	0.55 (0.40–0.75)	0.38 (0.23–0.64)	0.17	2.33 (1.91–2.84)	2.00 (1.44–2.76)	0.33
		65–74	1.77 (1.44–2.17)	1.67 (1.24–2.25)	0.09	6.15 (5.35–7.06)	4.19 (3.26–5.38)	1.96
		75–84	5.00 (4.20–5.95)	4.44 (3.43–5.74)	0.56	14.67 (12.95–16.59)	11.72 (9.46–14.48)	2.95
		85+	15.87 (12.54–19.99)	9.27 (5.83–14.57)	6.6	38.90 (32.34–46.26)	31.81 (22.41–43.89)	7.09
	Sex	female	1.97 (1.68–2.32)	1.71 (1.33–2.19)	0.26	6.46 (5.76–7.24)	5.39 (4.44–6.55)	1.07
		male	1.75 (1.51–2.03)	1.33 (1.05–1.68)	0.42	5.33 (4.79–5.93)	3.83 (3.17–4.63)	1.5
Death, ACS, stroke, heart failure	All		3.10 (2.83–3.39)	2.81 (2.46–3.21)	0.29	8.85 (8.28–9.45)	7.67 (6.88–8.55)	1.18
	Age	40–49	0.70 (0.42–1.17)	0.91 (0.48–1.73)	−0.21	1.69 (1.12–2.54)	1.87 (1.12–3.12)	−0.18
		50–64	1.40 (1.14–1.72)	1.73 (1.34–2.23)	−0.32	4.67 (4.05–5.39)	5.29 (4.32–6.47)	−0.62
		65–74	3.87 (3.32–4.51)	2.81 (2.19–3.61)	1.06	10.91 (9.78–12.17)	8.24 (6.76–10.02)	2.68
		75–84	7.42 (6.29–8.75)	6.86 (5.41–8.67)	0.57	20.64 (18.38–23.14)	16.43 (13.46–19.98)	4.21
		85+	19.91 (15.56–25.27)	16.25 (10.83–24.00)	3.66	45.53 (38.34–53.39)	36.60 (26.30–49.36)	8.93
	Sex	female	3.06 (2.67–3.51)	2.48 (2.00–3.08)	0.58	9.25 (8.38–10.20)	7.91 (6.67–9.36)	1.34
		male	3.12 (2.76–3.52)	3.04 (2.57–3.60)	0.07	8.54 (7.81–9.34)	7.50 (6.51–8.64)	1.04

## Discussion

This study found that there was a substantive heterogeneity between the populations using different classes of various diabetes medications and that statistical adjustment with the measured risk factors only partially eliminated this bias. Comparable populations used rosiglitazone and pioglitazone. Higher risks for death (overall and due to cardiovascular disease) and heart failure were found for rosiglitazone compared to pioglitazone, with the risks of death highest shortly after starting treatment and disappearing after discontinuation of rosiglitazone.

Several studies have evaluated the cardiovascular safety of different classes of diabetes medications [7]–[32]. As an example, Tzoulaki and collaegues concluded that sulphonylureas had an unfavourable risk profile compared with metformin [25]. All of these studies relied on regression analysis to deal with confounding but none tested whether this statistical approach indeed minimised confounding. Although not routinely conducted, the formal analysis of bias has been recommended to be a critical part of an analysis [36]. We used two approaches for this bias analysis (one evaluating diabetes patients to controls and one comparing past exposure of different drugs). Both these analyses suggested that statistical adjustment only partially removed confounding due to the underlying disease. But there may be alternative explanations of these findings, namely that most diabetes drugs have cardiovascular side-effects or that various diabetes drugs have persistent effects (increasing the risks during past exposure). Although we can not exclude with certainty these alternative explanations, the presence of residual confounding due to underlying disease seems most likely as in diabetes mellitus drug exposure is defined by diabetes severity. As drug exposure is defined by diabetes severity, epidemiological comparisons between different classes of diabetes medications should not simply rely on statistical adjustment with a few risk factors but should evaluate the extent of bias in these comparisons.

Rosiglitazone and pioglitazone appeared to be used in the UK by comparable populations, as indicated by the lack of differences in cardiovascular risks during past exposure and general similarity in baseline risk factors. Our findings of higher risks with rosiglitazone could be explained by a relatively higher level of toxicity or by a lower level of benefit with rosiglitazone. Given the challenges in comparing between different classes of diabetes medication, our study could not evaluate whether the findings are explained by excess toxicity or by lesser benefit with rosiglitazone. As an example, pioglitazone is well recognised to cause heart failure and the decreased RR of heart failure with pioglitazone may only indicate that pioglitazone is less toxic than rosiglitazone. The results of this study are consistent with those reported by Graham and collaegues using US Medicare data [31], although the follow-up in the present study was considerably longer. Also, we did not censor at a non-endpoint hospitalisation, as this could lead to differential loss to follow-up. A study by Wertz, that applied propensity matching to US claims data from insured employees, did not find any differences in the risk of death, MI and heart failure between rosiglitazone and pioglitazone [30]. Although this study appeared to be well conducted, the data concerned a restricted population with rates of death and MI lower than in the present study. Also, no results were provided without propensity score matching, which is a complex statistical technique. A recent meta-analysis of observational studies found a RR of mortality for rosiglitazone which were comparable to the present study, although it did report a small increased RR for myocardial infarction [37]. This meta-analysis did not adjust for data quality and consisted of pooling of studies with varying designs and study populations.

There is only indirect evidence from RCT comparing rosiglitazone and pioglitazone, as the large RCTs did not directly compare these drugs. But the RCTs do suggest differential effects. The RECORD study (comparing rosiglitazone with metformin or sulphonylurea to metformin plus sulphonylurea) found that the rate of cardiovascular death, MI and stroke was statistically comparable between the groups [38]. In contrast, the PROactive study found that this outcome was reduced with pioglitazone [39]. Similarly, the FDA meta-analysis found a trend for increased risks with rosiglitazone and decreased risks for pioglitazone (RRs of 2.14 and 0.54, respectively; P-values>0.05) [4]. The RRs for heart failure risks were also reported to be nominally higher with rosiglitazone than with pioglitazone [5]. These meta-analyses, lumping a set of heterogeneous studies, do not provide definitive evidence that rosiglitazone is inferior to pioglitazone. Observational studies, like the present one, are also limited by the potential for bias [39]. But despite the limitations of the present evidence base on the safety of rosiglitazone [40], an important consideration has been that there was limited evidence of a benefit of rosiglitazone on major clinical outcomes; the largest RCT (RECORD) did not show any beneficial effects of rosiglitazone [39]. The excess risks of cardiovascular outcomes in younger patients (under 65 years) was found to be small with rosiglitazone in this present study. Clearly, this finding of minimal adverse effects in younger patients needs be balanced by the lack of evidence of any beneficial effect of rosiglitazone in this group of patients.

There are significant challenges in interpreting the scientific evidence of the safety of medicines. Registration RCTs typically include a narrow set of patients recruited in specialised centres who are followed for a limited period of time. Often, these studies do not have the power to measure the effects on major clinical outcomes. Meta-analyses are now often conducted to overcome this limitation but the statistical lumping of heterogeneous RCTs is clearly not without limitations. The data from epidemiological studies often suffer from over-reliance on statistical adjustment and varying quality in data, design and study execution. Discrepant results within the same database are indicative of the methodological challenges in epidemiological research [41]. Also, there is often incomplete evidence after approval of a medicine about the targeting of the treatment and who should receive it. Thus, there is a major need to expand our toolbox for obtaining evidence on the effects of medicines. One option could be large simple RCTs conducted within a research database, allowing the clinician to randomise between treatments and then following for outcomes using the routine electronic health records.

There are various limitations to this study. Information on confounders and underlying disease severity was limited in this study. Furthermore, our analyses provide only simplistic representations of the actual exposures to diabetes medications. Drug exposure in actual clinical practice often varies greatly, with many different drug combinations being used and patients switching over time between drugs and patients being non-compliant to treatment instructions. We did not evaluate this complexity in exposure and also relied on information of prescriptions rather than actual use.

In conclusion, the findings in this study support the presence of unmeasured confounding in the comparisons of cardiovascular outcomes between different classes of diabetes medications due to heterogeneity in use (as reflected by the substantive differences in rates of death during past exposure). Comparable populations used rosiglitazone and pioglitazone. Higher risks for death (overall and due to cardiovascular disease) and heart failure were found for rosiglitazone compared to pioglitazone, with the risks of death highest shortly after starting treatment and disappearing after discontinuation of rosiglitazone. These excess risks were largest in patients aged 65 years or older. This study supports the suspension of rosiglitazone by European regulatory authorities in September 2010.

## Supporting Information

Appendix S1 Cumulative incidence (%) of ACS, stroke and heart failure (in GPRD) over one and three years in current rosiglitazone and pioglitazone users.
(DOC)
