# Correction: Risk of Death and Cardiovascular Outcomes with Thiazolidinediones: A Study with the General Practice Research Database and Secondary Care Data

**DOI:** 10.1371/annotation/8eb49a9f-06ae-4c79-bfe9-56974061321b

**Published:** 2013-02-25

**Authors:** Arlene M. Gallagher, Liam Smeeth, Suzie Seabroke, Hubert G. M. Leufkens, Tjeerd P. van Staa

The image for Figure 1 is incorrect. The correct image for Figure 1 can be seen here: 

**Figure:**
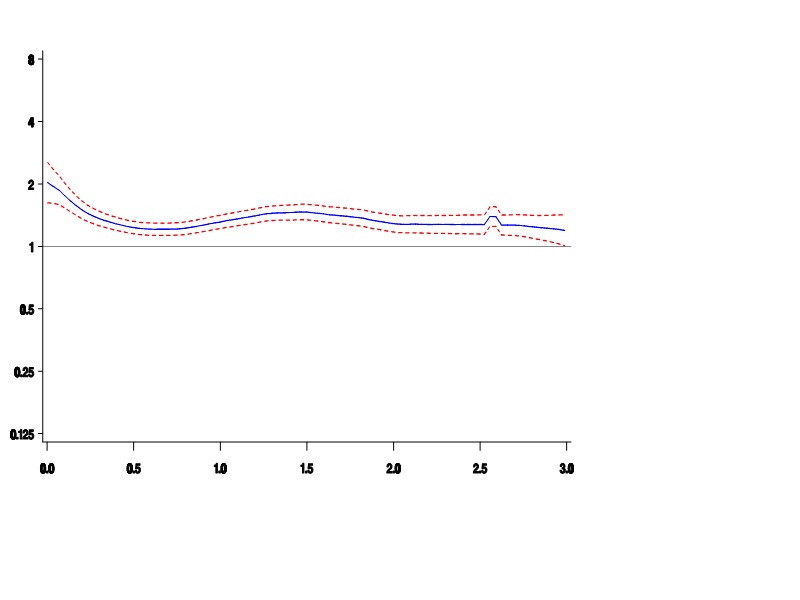


The Y axis label is 'Relative Rate', and the X axis label is 'Years of Treatment'. 

