# Supplementary material for: Risk of Death and Cardiovascular Outcomes with Thiazolidinediones: A Study with the General Practice Research Database and Secondary Care Data
Source: PLoS One. 2011 Dec 2;6(12):e28157. doi: 10.1371/journal.pone.0028157 (PMC3229530; doi:10.1371/journal.pone.0028157)
Supplement: Appendix S1 — Cumulative incidence (%) of ACS, stroke and heart failure (in GPRD) over one and three years in current rosiglitazone and pioglitazone users. (DOC) [file pone.0028157.s001.doc]

**Appendix S1.** Cumulative incidence (%) of ACS, stroke and heart failure (in GPRD) over one and three years in current rosiglitazone and pioglitazone users

|  |  |  |  | Year 1 | | |  |  | Year 3 | | |  |
| --- | --- | --- | --- | --- | --- | --- | --- | --- | --- | --- | --- | --- |
| Outcome | strata |  | Rosiglitazone | | Pioglitazone | Excess risk | | Rosiglitazone | | Pioglitazone | Excess risk | |
| ACS | All |  | 1.06 (0.91-1.24) | | 0.94 (0.75-1.18) | 0.12 | | 2.43 (2.15-2.75) | | 2.41 (1.99-2.92) | 0.02 | |
|  | Age | 40-49 | 0.36 (0.18-0.73) | | 0.71 (0.35-1.46) | -0.35 | | 0.51 (0.27-0.97) | | 1.04 (0.54-2.00) | -0.52 | |
|  |  | 50-64 | 0.64 (0.47-0.86) | | 1.01 (0.72-1.41) | -0.37 | | 1.66 (1.32-2.09) | | 2.46 (1.84-3.27) | -0.8 | |
|  |  | 65-74 | 1.55 (1.22-1.96) | | 0.72 (0.45-1.16) | 0.82 | | 3.17 (2.61-3.86) | | 1.75 (1.15-2.65) | 1.43 | |
|  |  | 75-84 | 1.89 (1.37-2.61) | | 1.38 (0.83-2.31) | 0.51 | | 4.73 (3.67-6.08) | | 4.33 (2.91-6.40) | 0.4 | |
|  |  | 85+ | 2.10 (0.93-4.72) | | 1.25 (0.30-5.18) | 0.84 | | 6.06 (3.31-10.98) | | 8.46 (3.10-21.94) | -2.4 | |
|  | Sex | female | 0.77 (0.59-1.01) | | 0.50 (0.31-0.81) | 0.27 | | 2.04 (1.65-2.52) | | 1.74 (1.19-2.56) | 0.3 | |
|  |  | male | 1.29 (1.07-1.55) | | 1.26 (0.98-1.63) | 0.03 | | 2.74 (2.35-3.19) | | 2.89 (2.32-3.61) | -0.16 | |
| Stroke | All |  | 0.55 (0.44-0.67) | | 0.38 (0.27-0.52) | 0.17 | | 1.23 (1.04-1.47) | | 0.99 (0.74-1.33) | 0.24 | |
|  | Age | 40-49 | 0.04 (0.01-0.26) | | 0.06 (0.01-0.41) | -0.02 | | 0.24 (0.07-0.83) | | 0.60 (0.21-1.72) | -0.36 | |
|  |  | 50-64 | 0.24 (0.15-0.39) | | 0.15 (0.07-0.35) | 0.09 | | 0.67 (0.46-0.97) | | 0.49 (0.26-0.94) | 0.18 | |
|  |  | 65-74 | 0.54 (0.36-0.79) | | 0.47 (0.27-0.79) | 0.07 | | 1.44 (1.07-1.95) | | 1.41 (0.88-2.24) | 0.03 | |
|  |  | 75-84 | 1.53 (1.10-2.14) | | 0.74 (0.39-1.40) | 0.79 | | 2.63 (1.93-3.60) | | 1.64 (0.87-3.06) | 1.0 | |
|  |  | 85+ | 3.79 (2.13-6.70) | | 3.86 (1.68-8.76) | -0.07 | | 8.17 (4.83-13.66) | | 3.86 (1.68-8.76) | 4.31 | |
|  | Sex | female | 0.56 (0.41-0.77) | | 0.44 (0.28-0.70) | 0.12 | | 1.32 (1.02-1.71) | | 1.23 (0.82-1.87) | 0.08 | |
|  |  | male | 0.54 (0.41-0.71) | | 0.33 (0.20-0.53) | 0.21 | | 1.18 (0.93-1.48) | | 0.83 (0.55-1.27) | 0.34 | |
| Heart failure | All |  | 0.82 (0.69-0.97) | | 0.77 (0.60-0.98) | 0.05 | | 2.51 (2.22-2.84) | | 2.13 (1.75-2.60) | 0.38 | |
|  | Age | 40-49 | 0.07 (0.02-0.30) | | 0 | 0.07 | | 0.23 (0.08-0.65) | | 0 | 0.23 | |
|  |  | 50-64 | 0.19 (0.11-0.33) | | 0.43 (0.26-0.72) | -0.24 | | 0.93 (0.68-1.26) | | 1.09 (0.71-1.68) | -0.16 | |
|  |  | 65-74 | 0.98 (0.74-1.30) | | 0.71 (0.44-1.14) | 0.27 | | 3.05 (2.49-3.73) | | 2.84 (2.03-3.96) | 0.21 | |
|  |  | 75-84 | 2.81 (2.19-3.60) | | 2.29 (1.57-3.34) | 0.52 | | 7.34 (6.04-8.90) | | 5.21 (3.81-7.11) | 2.12 | |
|  |  | 85+ | 3.23 (1.73-6.01) | | 3.93 (1.72-8.89) | -0.7 | | 12.87 (8.57-19.08) | | 6.55 (3.11-13.50) | 6.32 | |
|  | Sex | female | 1.01 (0.80-1.27) | | 0.56 (0.36-0.88) | 0.44 | | 3.00 (2.53-3.56) | | 2.08 (1.50-2.88) | 0.93 | |
|  |  | male | 0.67 (0.52-0.86) | | 0.91 (0.68-1.22) | -0.24 | | 2.15 (1.81-2.56) | | 2.17 (1.69-2.79) | -0.02 | |
